# Right Atrial Tumor Thrombus in Pediatric Nephroblastoma: A Case Report

**DOI:** 10.7759/cureus.108943

**Published:** 2026-05-16

**Authors:** Imane Chemlal, Houssain Benhaddou, Amal Bennani, El Mehdi Moutaouekkil, Ayad Ghanam

**Affiliations:** 1 Department of Pediatrics, Mohammed VI University Hospital, Faculty of Medicine and Pharmacy, Mohammed First University, Oujda, MAR; 2 Department of Pediatric Surgery, Mohammed VI University Hospital, Faculty of Medicine and Pharmacy, Mohammed First University, Oujda, MAR; 3 Department of Anatomopathology, Mohammed VI University Hospital, Faculty of Medicine and Pharmacy, Mohammed First University, Oujda, MAR; 4 Departement of Cardiovascular Surgery, Mohammed VI University Hospital, Faculty of Medicine and Pharmacy, Mohammed First University, Oujda, MAR

**Keywords:** atrium thrombus, nephroblastoma, pediatric case, right atrium tumor thrombus, wilms tumor

## Abstract

Nephroblastoma is the most common malignant renal tumor in children. Tumor thrombus extending into the vena cava and right atrium increases the complexity of surgical treatment. We report the case of a nine-year-old girl with nephroblastoma complicated by a tumor thrombus extending into the inferior vena cava and right atrium. She received four weeks of preoperative chemotherapy, which resulted in a reduction of both the primary tumor and the thrombus. The tumor thrombus was classified as T3c according to the 2010 AJCC staging system and as Level IV according to the Mayo Clinic classification. The surgical strategy was planned according to the extent of tumor thrombus as defined by these two classification systems. The patient then underwent en bloc resection of the tumor via a combined thoracoabdominal approach, consisting of an extended right nephrectomy via a subcostal incision with thrombectomy of the right atrium performed under extracorporeal circulation. Postoperatively, she received radiotherapy and additional chemotherapy based on risk stratification. At the three-year follow-up, she remains in complete remission with no evidence of recurrence. The presence of a tumor thrombus does not necessarily worsen prognosis if complete surgical resection is achieved. Preoperative chemotherapy can shrink the tumor and thrombus, facilitating resection and preventing complications. Simultaneous thoracoabdominal surgery under extracorporeal circulation enables en bloc resection of the primary tumor and thrombus. With multimodal treatment, children with Wilms tumor and tumor thrombus can achieve long-term survival.

## Introduction

Nephroblastoma, or Wilms tumor, is a kidney tumor that arises from primitive renal cells and accounts for more than 90% of kidney tumors in children [1,2]. It occurs predominantly in children aged one to five years, with a peak incidence around age three, accounting for approximately 75% of cases [2]. Globally, its incidence is estimated at 8 to 10 cases per 1,000,000 children [3]. This tumor is characterized by a marked tendency to invade the vasculature as a tumor thrombus, which can extend into the renal veins, the inferior vena cava, and, more rarely, the right atrium. Nevertheless, the presence of intravascular extension is not an adverse prognostic factor, although it reflects advanced disease requiring complex management [1,4].

We report a rare case of nephroblastoma complicated by a tumor thrombus in the right atrium in a nine-year-old girl who presented with an abdominal mass in the right flank, chronic abdominal pain, prolonged fever, hematuria, and vomiting. Based on this case, we further emphasize the importance of neoadjuvant chemotherapy to reduce tumor spread and optimize surgical management within a multidisciplinary team.

## Case presentation

A nine-year-old girl with no significant past medical history presented to our pediatric oncology department for evaluation of chronic right-sided abdominal pain persisting for five months, accompanied by intermittent vomiting, prolonged fever, and hematuria over the past two weeks. Physical examination revealed a hard, painless abdominal mass in the right flank and fixed to the deep planes. Microscopic hematuria was detected on a urine dipstick test, with no signs of urinary tract infection. 

**Figure 1:**
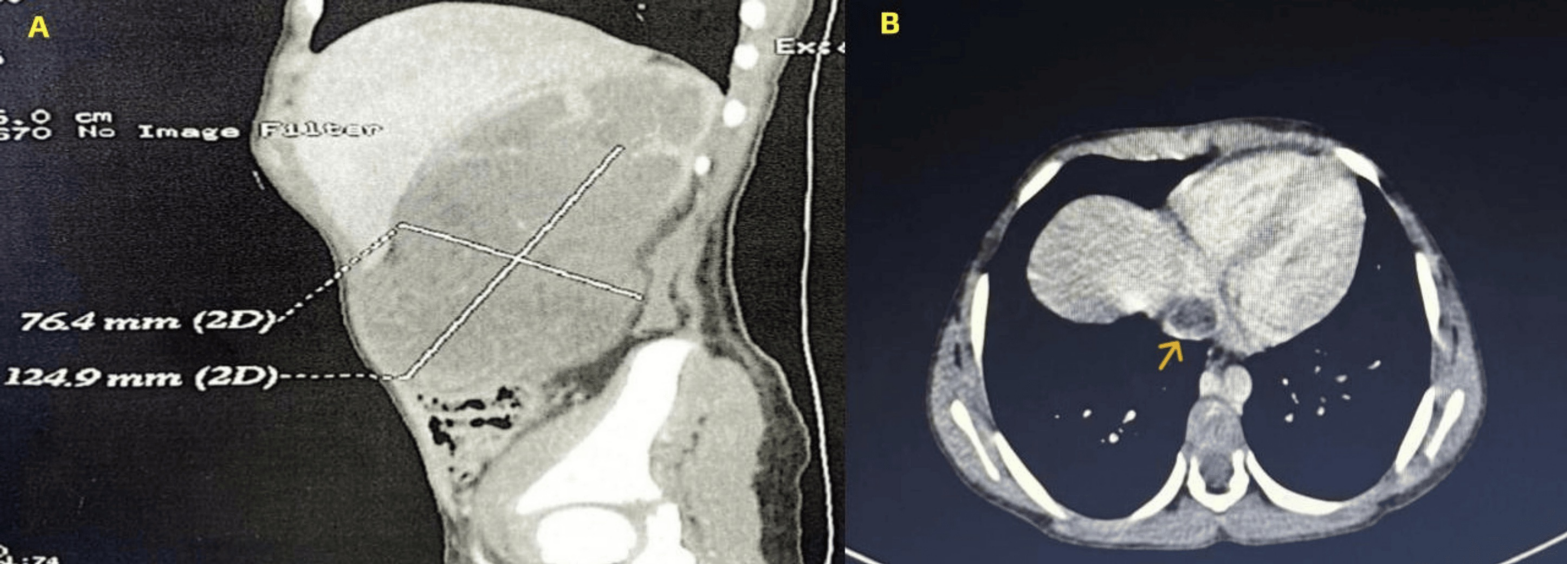
Thoraco-abdominopelvic computed tomography scan showing a nephroblastoma complicated by an inferior vena cava thrombus extending into the right atrium. A: Sagittal section of an abdominopelvic computed tomography scan showing a tissue mass occupying the lower two-thirds of the right kidney, consistent with nephroblastoma. B: Axial contrast-enhanced chest computed tomography scan showing a hypodense intraluminal material consistent with a thrombus in the inferior vena cava (arrow), extending up to its junction with the right atrium.

**Figure 2:**
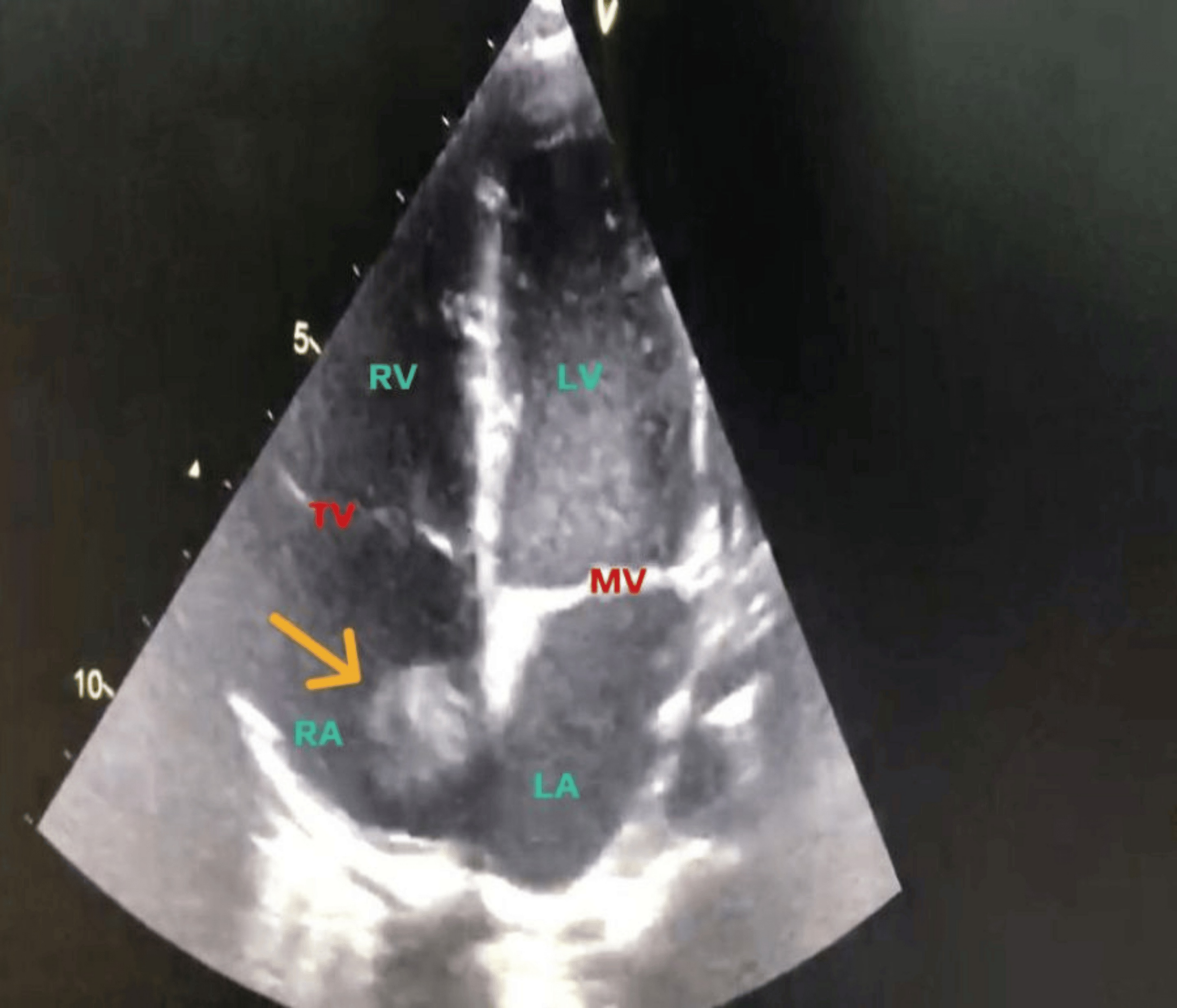
2D echocardiography showing a right atrial thrombus (arrow). RA: right atrium; LA: left atrium; RV: right ventricle; LV: left ventricle; TV: tricuspid valve; MV: mitral valve

Abdominal ultrasound, complemented by thoraco-abdominopelvic computed tomography, revealed a mass occupying the lower two-thirds of the right kidney, measuring 124 × 76 x 67 mm in its largest dimensions, consistent with nephroblastoma (Figure 1A). Associated thrombosis of the right renal vein extended into the inferior vena cava and reached the right atrium (Figure 1B). This was also confirmed by two-dimensional echocardiography (Figure 2). Laboratory findings (Table 1) revealed normochromic, normocytic anemia with an elevated lactate dehydrogenase level. The remainder of the workup, including renal function tests, serum electrolytes, liver function tests, and urinary catecholamines, was within normal limits.

**Table 1:** Summary of the patient’s laboratory findings. Hb: hemoglobin; Hct: hematocrit; MCV: mean corpuscular volume; MCH: mean corpuscular hemoglobin; MCHC: mean corpuscular hemoglobin concentration; PLT: platelets; WBC: white blood cell count; ANC: absolute neutrophil count; LDH: lactate dehydrogenase; AST: aspartate aminotransferase; ALT: alanine aminotransferase; ALP: alkaline phosphatase; VMA: vanillylmandelic acid; HVA: homovanillic acid

Parameters	Result	Reference Range
Hb	9.0 g/dl	12-16 g/dl
Hct	26.30%	37-47%
MCV	84,00 fl	80,00-98,00 fl
MCH	28.80 pg	27,00-32,00 pg
MCHC	34.20%	32,00-36,00%
PLT	408,000 cells/µl	150,000-400,000 cells/µl
WBC	9,470 cells/µl	4,000 -10,000 cells/µl
ANC	6,520 cells/µl	1,500-7,000 cells/µl
Lymphocytes	1,900 cells/µl	1,000-4,000 cells/µl
Monocytes	590 cells/µl	200-800 cells/µl
Eosinophils	400 cells/µl	0-500 cells/µl
Basophils	60 cells/µl	0-200 /µl
Urea	0.29 g/l	0.10-0.30 g/l
Creatinine	6.97 mg/l	5.7-11.1 mg/l
Serum sodium	139 mEq/l	138-145 mEq/l
Serum potassium	4.0 mEq/l	3.4-4.7 mEq/l
Serum calcium	89 mg/l	84-102 mg/l
LDH	828 UI/l	125-243 UI/l
Uric acid	36.50 mg/l	26-60 mg/l
AST	19 UI/l	5-34 UI/l
ALT	8 UI/l	0-55 UI/l
GGT	12 UI/l	9-36 UI/l
ALP	100 UI/l	< 500 UI/l
Adrenaline	< 0.02 µmol/l	< 0.02 µmol/l
Noradrenaline	0.05 µmol/24h	< 0.50 µmol/24h
Dopamine	0.07 µmol/mmol	< 0.80 µmol/mmol
HVA	5.42 µmol/mmol	< 14,00 µmol/mmol
VMA	3.57 µmol/mmol	< 10,00 µmol/mmol

The patient subsequently received four cycles of preoperative chemotherapy based on actinomycin D (45 γ/kg) combined with vincristine (1.5 mg/m^3^). After one month, a thoraco-abdominopelvic computed tomography scan was performed, showing a 21% reduction in the size of the right renal tumor, with regression of the tumor thrombus.

**Figure 3:**
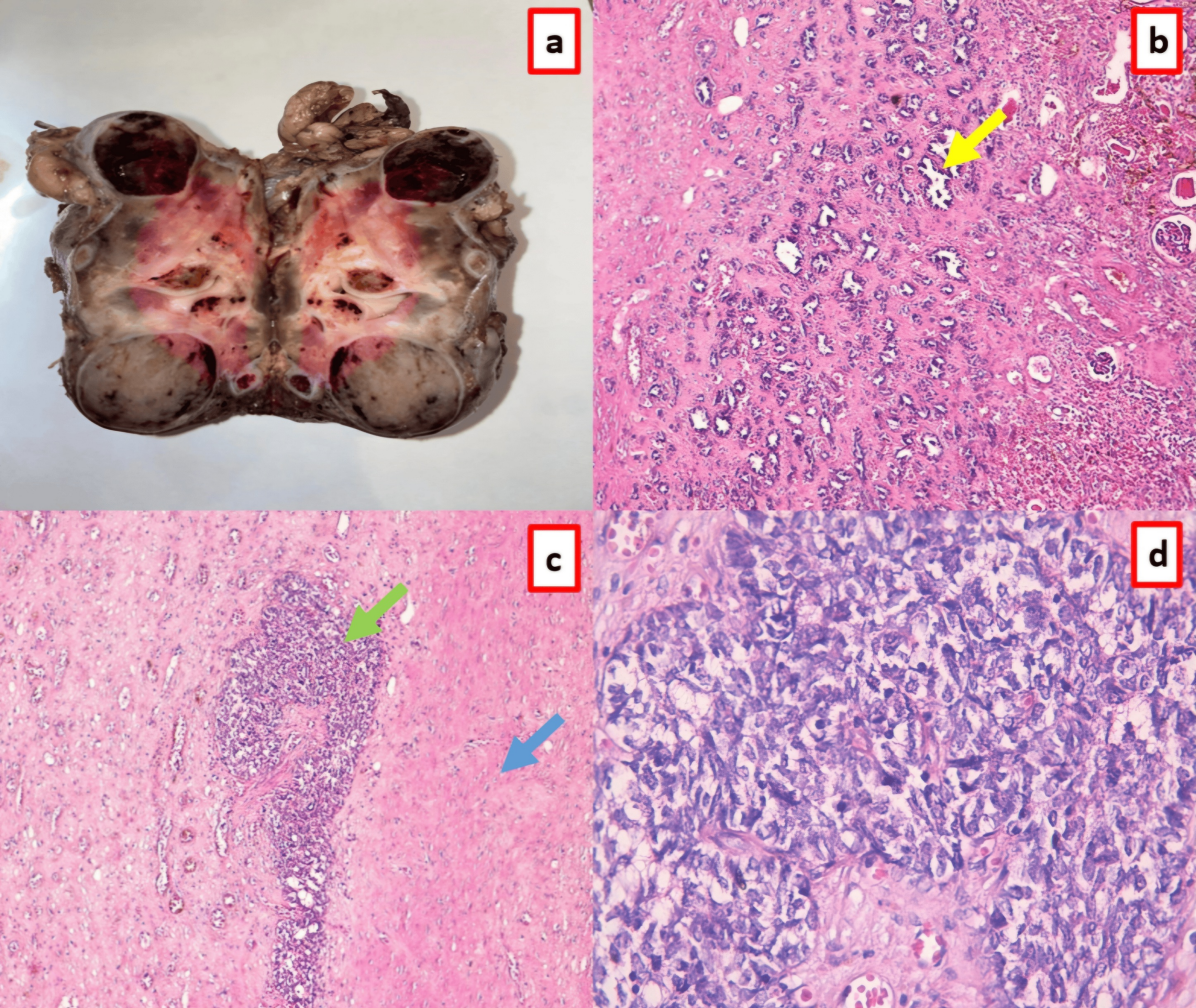
Macroscopic appearance and histological examination of the surgical specimen. (a) Macroscopic appearance of a sagittal section of a kidney, largely replaced by a centrally located, ill-defined, extensively altered whitish mass. Histological analysis reveals a mixed nephroblastoma composed of: (b) an epithelial component (yellow arrow), (c) a mesenchymal component (blue arrow) and a blastemal component (green arrow). (d) The blastemal component consists of small round cells with high mitotic activity. H&E: Hematoxylin and eosin staining. (b, c): H&E ×100; (d): H&E ×400

**Figure 4:**
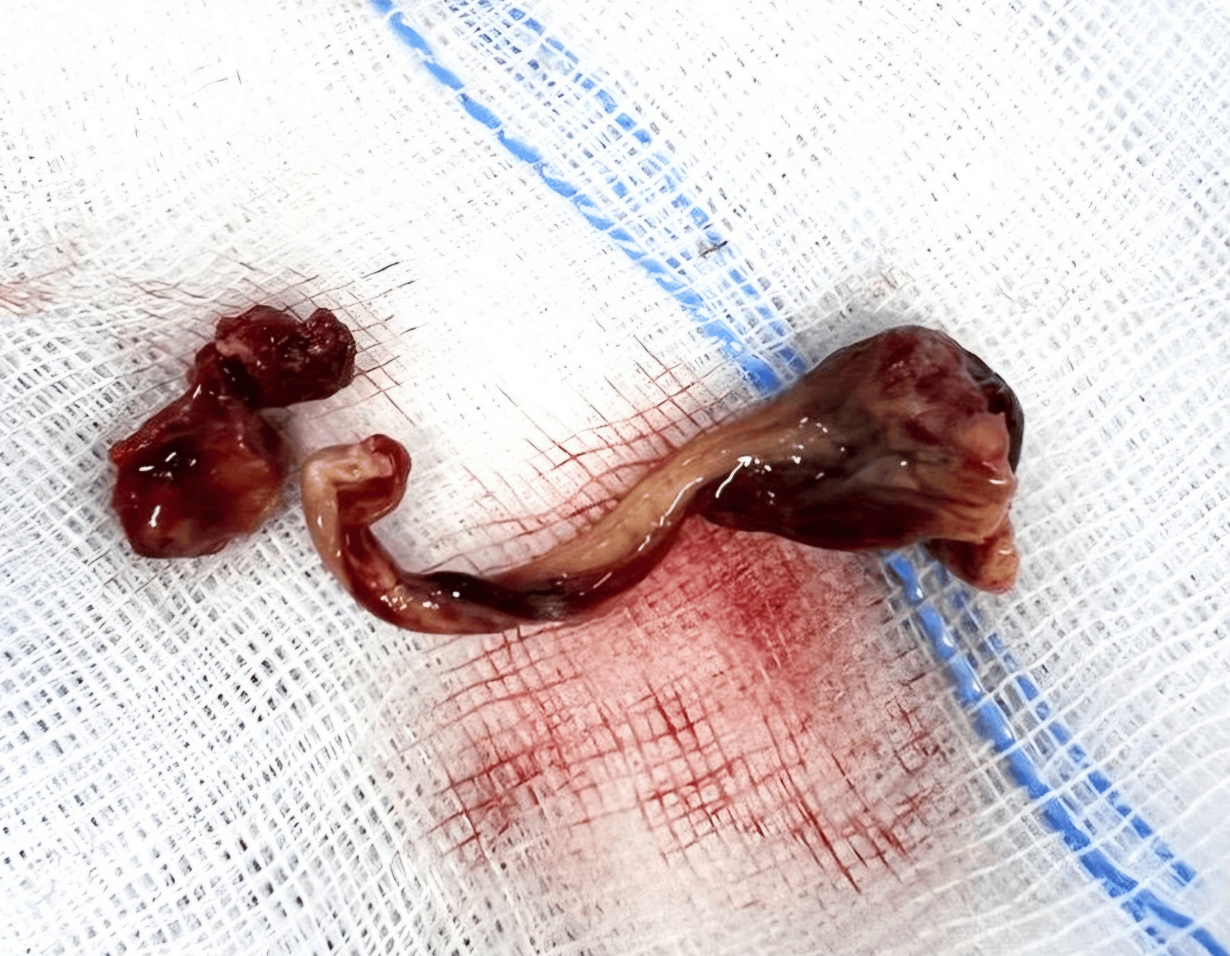
Right atrial thrombus removed during thrombectomy.

The patient underwent an en bloc extended right nephrectomy (Figure 3a) via a subcostal approach, with thrombectomy of the right atrium (Figure 4) performed under extracorporeal circulation. Cardiopulmonary bypass was performed under normothermic conditions. Right atriotomy was performed parallel to the right atrioventricular groove, revealing a tumor thrombus extending from the inferior vena cava into the right atrium. A brief (<1 minute) interruption of cardiopulmonary bypass was required to perform thrombectomy, followed by immediate reinstatement of extracorporeal circulation. Cardiopulmonary bypass support lasted 60 minutes. Histopathological examination of the surgical specimen confirmed a mixed-type nephroblastoma (Figures 3b-3d). She then received radiotherapy for three weeks, concurrently with adjuvant chemotherapy consisting of etoposide (150 mg/m^3^) and carboplatin (200 mg/m^2^), alternating with cyclophosphamide (450 mg/m^3^) and doxorubicin (50mg/m^2^), for 34 weeks, with a favorable outcome.

**Figure 5:**
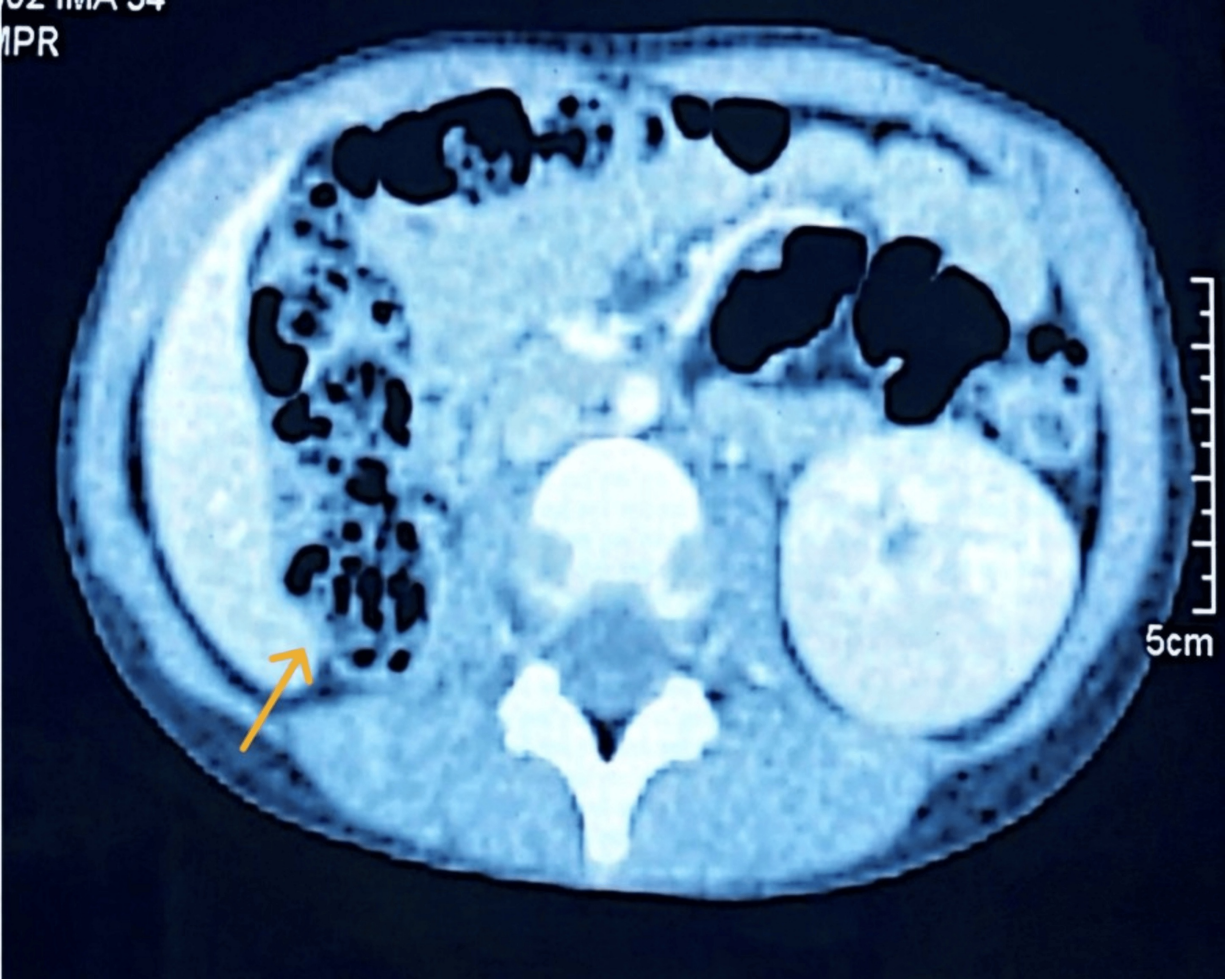
Sagittal contrast-enhanced abdominal CT scan showing an empty right renal fossa (arrow) without evidence of disease recurrence or progression. CT: Computed tomography

One year after diagnosis, a cerebro-cervico-thoraco-abdomino-pelvic computed tomography scan showed an empty right renal fossa, complete resolution of thrombosis, with no evidence of disease progression in any of the evaluated regions (Figure 5). The clinical course was favorable, and at three-year follow-up, the patient remains in complete remission.

## Discussion

Nephroblastoma is the most common malignant kidney tumor in children [2] and is the second most common pediatric tumor in Morocco, accounting for 15.8%, after acute lymphoblastic leukemia [5]. It results from abnormalities in renal embryonic development associated with genetic alterations [2]. A slight female predominance has been reported [6]. Prognosis is generally favorable, with five-year survival rates exceeding 90% in high-income countries and less than 50% in low and middle-income countries [7]. 

The prognosis of Wilms tumor depends mainly on the tumor stage, histological subtype, genetic alterations, and response to treatment [8]. Advanced disease with metastases and unfavorable histological features, such as anaplasia and post-chemotherapy blastemal type, are associated with poorer outcomes [8]. In addition, several genetic, protein, and immunologic biomarkers have been identified and are strongly associated with recurrence risk, including loss of heterozygosity at 1p, 16q, and 11p15, gain of 1q, and TP53 mutations [9]. Finally, poor therapeutic response and residual disease after surgery remain important adverse prognostic factors [8]. 

Tumor extension into the inferior vena cava occurs in 4% to 10% of cases, whereas right atrial involvement remains rare, with an estimated prevalence of 0.7% to 3.4% [1,10]. This extension most often begins in the right renal vein (59% to 85% of cases), likely because it is shorter than the left renal vein [1]. In some cases, the thrombus may progress to the tricuspid valve or even extend into the right ventricle [10]. In our series of nephroblastoma cases, only one case of a right atrial thrombus was observed, confirming the rarity of this location. These findings are consistent with the literature. Abdullah et al. report a prevalence of 3.4% for right atrial thrombi [10], while Talat et al. report a prevalence of 1.6% [4]. 

In most cases, nephroblastoma is asymptomatic and discovered incidentally. In fewer than 25% of cases, it is symptomatic, with hematuria, hypertension, or flank pain as the main presentations [6]. Similarly, intracardiac thrombi are most often detected incidentally on imaging studies and are typically asymptomatic. For this reason, systematic evaluation for intracardiac extension of the thrombus is recommended in all patients with nephroblastoma [11]. In our patient, who presented with abdominal pain, vomiting, and hematuria but no hypertension, a right atrial thrombus was incidentally discovered on cardiac ultrasound and chest computed tomography.

The diagnosis of nephroblastoma is based primarily on clinical and radiologic findings. Abdominal ultrasound is the initial imaging modality, often followed by computed tomography or magnetic resonance imaging to assess tumor extent and stage the disease for treatment planning [6]. These advanced imaging techniques also enable accurate detection of intravascular and intracardiac tumor thrombi, providing detailed information on their location, size, and relationship to the vascular wall [11]. In cases of caval tumor thrombus, progression toward the right atrium is common, making Doppler ultrasound essential for assessing blood flow before and after treatment [4]. To minimize the risk of tumor spread, a preoperative biopsy is not routinely indicated, particularly for resectable unilateral renal tumors [2].

The management of nephroblastoma requires a multidisciplinary approach based on disease stage and histology, combining surgery, chemotherapy, and radiotherapy [6]. Tumor thrombus extension above the suprahepatic veins is a clear contraindication to initial surgery and necessitates neoadjuvant chemotherapy [2].

Neoadjuvant chemotherapy combining vincristine and actinomycin D is typically administered for 4 to 8 weeks [4]. It helps reduce tumor volume and thrombus size, thereby facilitating surgical resection. A favorable response is observed in most cases, including those with the inferior vena cava and right atrial involvement [1], which helps reduce the risk of surgical complications [12]. Talat et al. reported thrombus regression in 70.5% of cases, with complete resolution in 5.8%, and a decrease in the prevalence of right atrial thrombi from 17.6% to 11.7% following neoadjuvant chemotherapy [4]. In addition, Naik-Mathuria et al. reported that chemotherapy led to regression of intracardiac thrombus in 45% of cases and was associated with a significantly lower rate of perioperative complications when used as neoadjuvant treatment (25%) compared with upfront surgery (55%) [13]. In our case, the patient received preoperative chemotherapy with vincristine and actinomycin D for four weeks, resulting in a favorable response, with marked regression of the tumor thrombus on follow-up imaging one month later.

Thrombus regression after chemotherapy may allow avoidance of cardiopulmonary bypass when the thrombus remains subdiaphragmatic, enabling removal via venotomy with control of the inferior vena cava. Conversely, in cases of atrial extension or parietal adhesion, more extensive surgery is required, including an atriotomy under cardiopulmonary bypass, sometimes combined with a caval thrombectomy or even resection of the inferior vena cava. In extensive forms, the use of cardiopulmonary bypass with circulatory arrest under deep hypothermia allows complete excision in a bloodless surgical field, with the possibility of vascular reconstruction and, in some cases, a two-stage approach [10]. 

In these complex situations, optimal management requires the involvement of cardiothoracic surgeons, as cardiopulmonary bypass is essential for safe intracardiac tumor thrombus extraction. In addition, transesophageal echocardiography plays a crucial role by enabling real-time assessment of tumor extension and its potential adherence to the myocardium and tricuspid valve, thereby guiding the decision regarding the necessity of atriotomy, as previously described by Karnes and Blute [14].

Following surgical treatment, including total nephrectomy, adjuvant therapy for Wilms tumor consists of chemotherapy and radiation therapy, tailored to histopathological classification and postoperative clinical stage [11]. In advanced stages, radiotherapy plays an especially important role, and early initiation, preferably within 10 days after surgery, may improve outcomes. This standardized multimodal strategy has significantly improved long-term survival in most pediatric patients [11]. Our patient received multimodal treatment and achieved complete remission at three years of follow-up without recurrence. This suggests that intracardiac extension, although rare and severe in appearance, may not necessarily worsen prognosis when complete surgical resection and appropriate multimodal therapy are achieved.

## Conclusions

Tumor thrombus occurs in several cancers, mainly Wilms tumor, renal cell carcinoma, adrenocortical carcinoma, and hepatocellular carcinoma, but may also be observed in other benign or malignant conditions. Imaging is key for diagnosis and staging, and its presence indicates a poorer prognosis and significantly influences treatment strategy. Atrial tumor thrombus is a rare but challenging manifestation of nephroblastoma that requires complex, carefully planned management. Our case highlights that early recognition of vascular extension, appropriate use of neoadjuvant chemotherapy, and coordinated surgical management are essential to reduce operative risks and improve outcomes. As demonstrated by our patient, long-term remission is achievable even in advanced presentations when complete resection and risk-adapted adjuvant therapy are successfully implemented.
